# Recurrent cementoblastoma with multifocal growth and cellular atypia: a case report

**DOI:** 10.1186/s13000-024-01479-0

**Published:** 2024-04-08

**Authors:** Kaori Oya, Akinori Takeshita, Kanta Wakamori, Misa Song, Hayato Kimura, Katsutoshi Hirose, Hiroaki Shimamoto, Sunao Sato, Kazuhide Matsunaga, Narikazu Uzawa, Satoru Toyosawa

**Affiliations:** 1 Division of Clinical Laboratory, Osaka University Dental Hospital, Suita, Osaka Japan; 2 Department of Oral and Maxillofacial Oncology and Surgery, Osaka University Graduate School of Dentistry, Suita, Osaka Japan; 3 Department of Diagnostic Pathology, Itami City Hospital, Itami, Hyogo Japan; 4 Department of Oral and Maxillofacial Pathology, Osaka University Graduate School of Dentistry, Suita, Osaka Japan; 5 Department of Oral and Maxillofacial Radiology, Osaka University Graduate School of Dentistry, Suita, Osaka Japan

**Keywords:** Odontogenic tumor, Cementoblastoma, Recurrence, Cellular atypia, c-FOS

## Abstract

**Background:**

Cementoblastoma is a rare odontogenic tumor characterized by the formation of osteocementum-like tissue on a tooth root directly by neoplastic cementoblasts. Although it is categorized as benign, it has a high potential for growth with a certain degree of recurrence risk. However, there are only a few studies describing the features of recurrent cementoblastoma. The diagnosis of recurrent cementoblastoma is challenging not only due to its cytological atypia but also because of its large size and multicentric growth pattern. These characteristics suggest a potential for malignancy.

**Case presentation:**

A 29-year-old woman was transferred to our university dental hospital complaining of swelling of the right mandible. She had a history of enucleation of cementoblastoma associated with the third molar of the right mandible. Five years after the initial treatment, imaging demonstrated well-circumscribed multicentric radiopaque lesions in the same area. Histologically, the lesion consisted of osteocementum-like tissue rimmed with polygonal or plump tumor cells. Several cells were large epithelioid cells with bizarre nucleoli, which may be reminiscent of malignant tumors. Otherwise, there were no apparent malignant findings, including proliferative activity or atypical mitotic figure. Besides, tumor cells were positive for c-FOS, a marker of osteoblastoma and cementoblastoma. Eventually, the patient was diagnosed with recurrent cementoblastoma.

**Conclusions:**

Pathological analyses of this case suggested that the recurrent event in the cementoblastoma altered its growth pattern and tumor cell shape. Moreover, in the case of enucleation surgery, long-term follow-up is important because there is some recurrent risk of cementoblastoma, although it is not high.

## Background

Cementoblastoma is a distinctive benign neoplasm that originates from odontogenic ectomesenchyme. It is characterized by the formation of osteocementum-like tissue, which is deposited directly on a tooth root by neoplastic cementoblasts [1]. Although it is a benign tumor, it has a high potential for growth [2, 3], with a certain degree of recurrence risk [3–5]. The reported recurrence rate of cementoblastoma varies from 11.8% [4] to 37.1% [3].

Histologically, tumor margins with radiating trabeculae rimmed by plump cementoblasts are characteristically observed [1]. However, cementoblastomas are difficult to differentiate from osteoblastoma because of the morphological analogy between them [6]. Slootweg [6] concluded that cementoblastomas and osteoblastomas have the same histological appearance; therefore, the diagnosis of cementoblastoma should not be made unless the lesion is connected with a tooth. Jelic et al. [2] also reported that the cementum is virtually indistinguishable from bone. Additionally, the possibility of osteosarcoma should be considered in differential diagnosis because neoplastic cementoblasts are often quite pleomorphic [7, 8].

Herein, we present a case of recurrent cementoblastoma using radiological and histological data to better understand the lesion.

## Case presentation

**Fig. 1:**
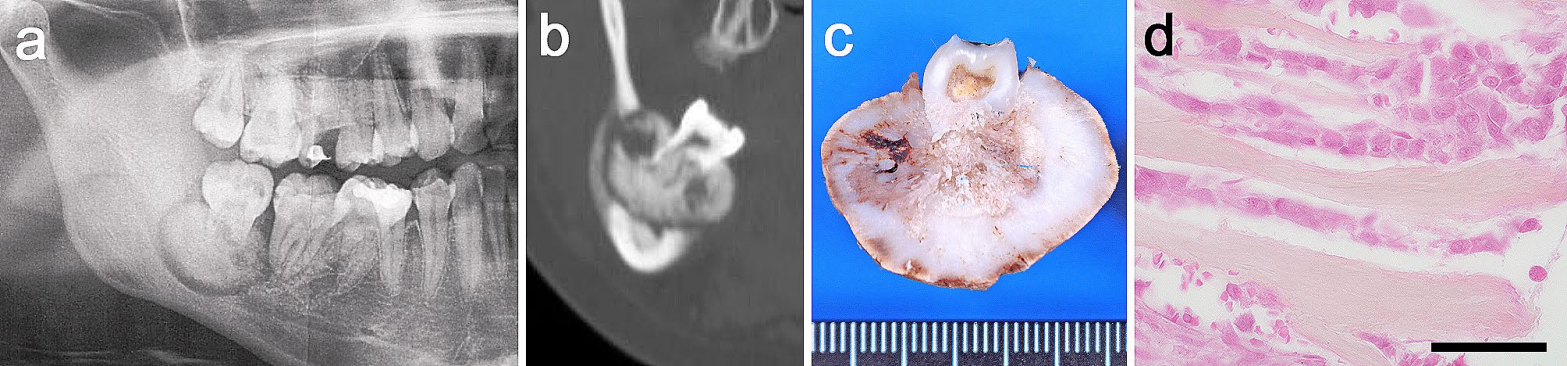
Imaging, gross appearance, and histopathological findings of primary lesion. **(a)** Panoramic radiograph and **(b)** coronal cone-beam computed tomography image show a radiopaque mass with a uniform radiolucent rim, including the root of a third molar in the right mandible. Expansion and perforation of cortical bone were observed. **(c)** The gross appearance of the primary lesion is divided into halves. The mass was fused to the tooth root. **(d)** Although the staining property was not good due to over-decalcification, cementoblast-like plump cells around the rim of radiated hard tissue are observed (Hematoxylin and eosin staining, 40×) scale bar: 50 μm

A 29-year-old woman was transferred to our university dental hospital complaining of swelling of the right mandible. She had a history of enucleation of cementoblastoma associated with the third molar at the same site 5 years previously **(**Fig. 1**)**. A panoramic radiograph showed a radiopaque mass fused to the tooth root and surrounded by a clear radiolucent rim **(**Fig. 1a**)**. Cone-beam computed tomography (CBCT) demonstrated expansion and perforation of the cortical bone **(**Fig. 1b**).** Macroscopically, the adhesion of the mass to the tooth root was confirmed **(**Fig. 1c**)**. Histologically, although the staining property was not good due to over-decalcification, cementoblast-like plump cells around the rim of radiated hard tissue were observed **(**Fig. 1d**)**.

**Fig. 2:**
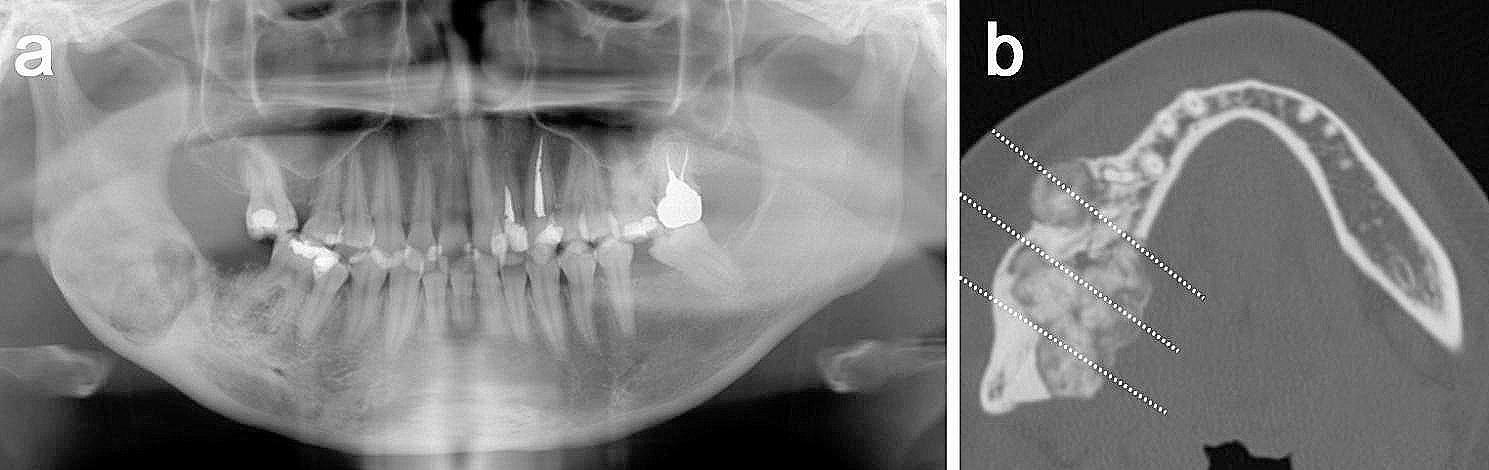
Imaging findings of recurrent lesion. **(a)** Panoramic radiograph revealed a large, radiopaque lesion in the postoperative region. **(b)** Axial cone-beam computed tomography image showed multicentric masses surrounded by a thin radiolucent zone. Dotted lines indicate the site of the cut surface, as shown in Fig. 3

In the current lesion, a panoramic radiograph showed a large mass with heterogeneous radiopacity in the edentulous postoperative region **(**Fig. 2a**)**. CBCT images demonstrated multicentric masses, measuring 12 × 11 × 10 mm (buccal side) and 29 × 35 × 21 mm (lingual side), surrounded by thin radiolucent rims **(**Fig. 2b**)**. Expansion, thinning, and perforation of the cortical bone were observed. These clinical and radiographic findings suggested a possibility of recurrent cementoblastoma. However, a suspicion for other possibilities, including malignancy, remained histologically due to the insufficient sampling of the incisional biopsy, which contained only a few atypical plump cells.

**Fig. 3:**
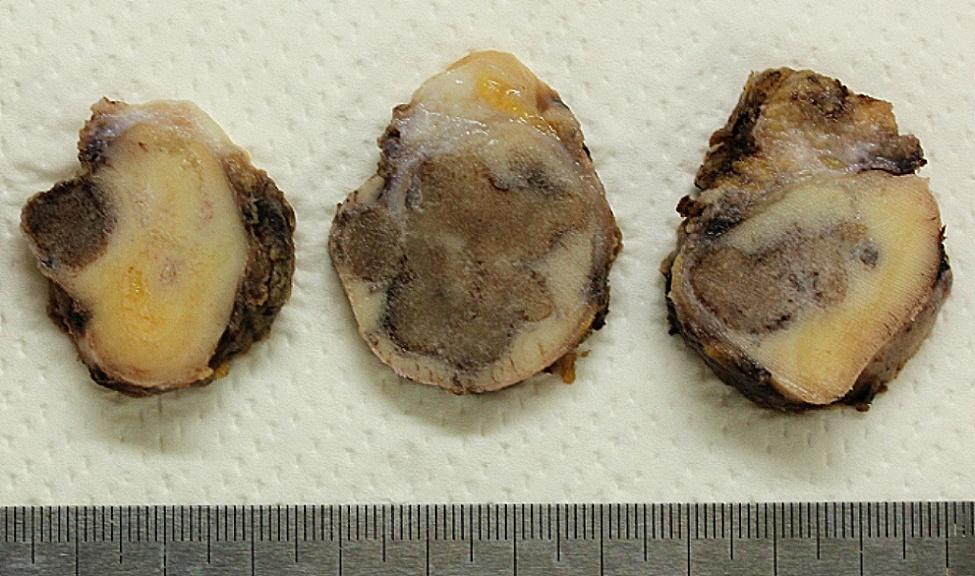
Gross appearance of the recurrent lesion. Well-circumscribed bone-like hard masses are observed on the cut surface.

**Fig. 4:**
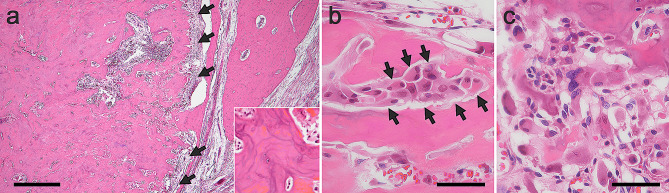
Histopathological findings. **(a)** Paraffin section (hematoxylin and eosin) revealed that the lesion mainly consists of osteocementum-like hard tissue with basophilic, irregular reversal lines (inset). Thin fibrous tissue (arrow) is observed between the lesion and surrounding tissue (4×). **(b)** The hard tissue was rimmed with plump cells (arrow, 40×). **(c)** The cellular atypia of tumoral cells was often outstanding (40×). Scale bar: a: 500 μm; b, c: 50 μm

**Fig. 5:**
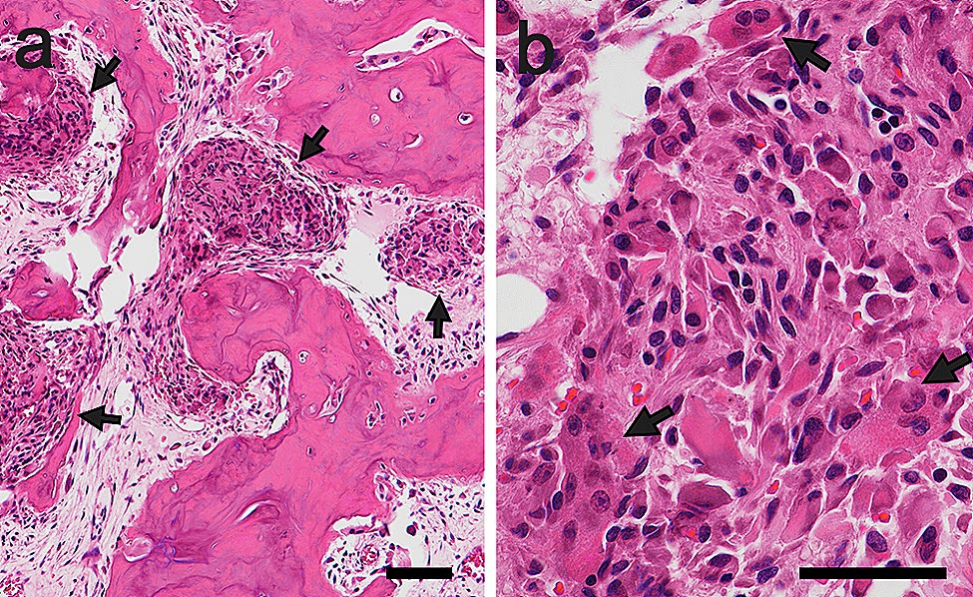
Aggregation of cells. **(a)** In the soft tissue dominant area, there are several clusters consisting of aggregated atypical cells (arrow, 10×) and **(b)** multinucleated cells (arrow, 40×). Scale bar: a: 100 μm; b: 50 μm

Under general anesthesia, mandibular segmentectomy and reconstruction using a scapular flap were performed, and the specimen was subjected to histopathological examination. After formalin fixation, the lesion macroscopically appeared as a well-demarcated, brown, and bony hard mass **(**Fig. 3**)**. Histological examination with hematoxylin and eosin staining revealed that the lesion mainly consisted of osteocementum-like hard tissue with basophilic, irregular reversal lines. The outer border of the masses was well-defined; thin fibrous tissue was observed between the lesion and the surrounding tissue despite the presence of cortical bone perforation. **(**Fig. 4a**)**. The hard tissue was rimmed with osteoblast- or cementoblast-like polygonal or plump tumor cells **(**Fig. 4b**)**. The tumor-cell size ranges from 10 to 30 μm in width. Some of the plump cells were binucleated. The cellular atypia of tumoral cells, showing anisokaryosis, hyperchromasia, and bizarre nuclei, was often outstanding, but mitosis was not observed. **(**Fig. 4c**)**. The osteoclast-like multinucleated cells were scattered. There were several clusters consisting of aggregated atypical cells and multinucleated cells **(**Fig. 5a, b**)**. Tumor infiltration into the surrounding tissue was not observed. The fibrous tissue between trabeculae included rich blood vessels.

**Fig. 6:**
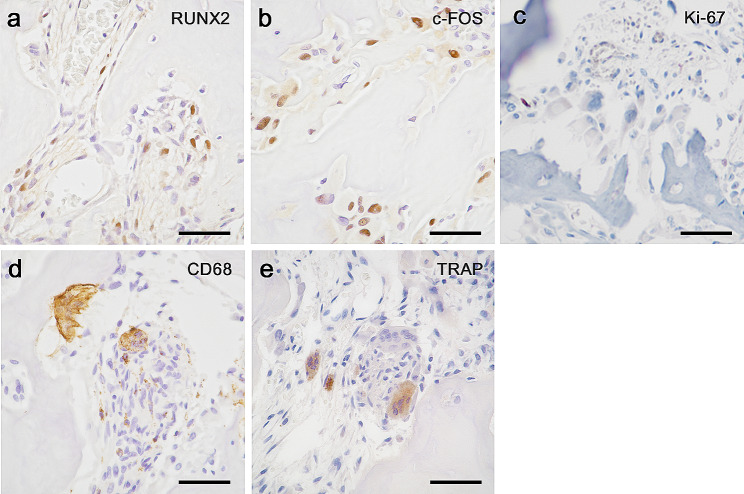
Immunohistochemical staining results for **(a)** RUNX2, **(b)** c-FOS, **(c)** Ki-67, **(d)** CD68, and **(e)** TRAP (40×). Scale bar: 50 μm

Immunostaining for RUNX2, a marker of the cells in both osteogenesis and cementogenesis, was positive in some spindle and small tumor cells. Tumor cells were positive for c-FOS, a marker of osteoblastoma and cementoblastoma **(**Fig. 6a, b**)**. Ki-67 immunostaining revealed its low proliferative activity (< 1%) **(**Fig. 6c**)**. Immunostaining results for MDM2 and CDK4 were negative. These results were consistent with those for benign tumors. The multinucleated cells were positive for CD68 and TRAP **(**Fig. 6d, e**)**. Atypical cells in the clusters were partially weakly positive for c-FOS but negative for RUNX2, CD68, and TRAP.

A final diagnosis of recurrent cementoblastoma was made based on radiological and histological findings.

A follow-up CT scan performed 7 months postoperatively showed no evidence of recurrence, and the patient is making satisfactory progress. A half-yearly follow-up has been scheduled.

## Discussion and conclusions

The differential diagnosis for recurrent cementoblastoma includes benign to malignant disease because of its large size, multicentric growth pattern, and cytological atypia [3, 5, 7, 9].

Histologically, it is necessary to rule out osteosarcoma due to cellular atypia, such as anisokaryosis and hyperchromasia, for treatment planning and prognosis prediction [7–9]. The following information may help in the histopathological diagnosis. First, there is no definitive immunohistochemical marker for conventional osteosarcoma; however, it expresses broad markers, such as S100, EMA, and keratin [10], and shows a high proliferative activity [11], as evident from the high Ki-67 positive rate. On the other hand, in cementoblastomas, tumor cells show low proliferative rates and rare mitotic activities, suggesting their slow-growing property [1, 7]. Second, rearrangement of *FOS* and *FOSB* and *c-FOS* overexpression using an antibody for the N-terminus of *c-FOS* was reported in osteoid osteoma, osteoblastoma, and cementoblastoma recently [12–14]. *c-FOS* expression was identified in osteosarcomas more than two decades ago; however, a significant proportion of osteosarcomas did not exhibit c-FOS immunopositivity, as evidenced by antibodies targeting the N-terminus of c-FOS [13, 15]. It is possible that the antibodies utilized in earlier studies recognized epitopes within the protein distinct from those identified by the current antibody [13]. c-FOS immunostaining has proven useful in diagnosing osteoid osteoma, osteoblastoma, and cementoblastoma, particularly when overexpression is observed in the majority of tumor cells [13–15]. Despite a small percentage of osteosarcoma (4–14%) demonstrating *c-FOS* expression [13, 15], the expression tends to be focal, with immunoreactivity predominantly observed in nonosteoblastic areas [13]. Third, MDM2/CDK4 co-expression is specific to osteosarcoma, which progressed from low-grade central osteosarcoma, even though MDM2/CDK4 expression is not common in conventional osteosarcoma [16, 17]. In the present case, the low proliferative activity of tumor cells was confirmed by Ki-67 immunostaining; mitosis was not observed. Cells were positive for c-FOS and negative for MDM2 and CDK4 immunostaining. These results reduce the possibility of malignancy and are consistent with those for benign tumors.

It is difficult to discriminate between cementoblastoma and osteoblastoma based solely on the morphological features described above [6]. The similarity in their component proteins further complicates their differentiation [18]. In addition, a common genetic feature has been identified: *FOS* or *FOSB* rearrangement [12–14].

At present, radiological findings provide convincing evidence for diagnosis. Radiologically, our case was consistent with cementoblastoma, which typically manifests as a well-defined radiopaque mass with a thin and uniform radiolucent border [3, 5]. Recurrent cementoblastomas can form multiple central masses, although there are only three English-language literature describing the feature (Table 1) [5, 19, 20]. Additionally, expansion, erosion, or perforation of the bony cortex could be observed in recurrent cementoblastoma [3, 5]. Osteoblastomas show a more irregular pattern of radiopacity than cementoblastoma [3]; the lesion may be surrounded by reactive sclerosis [21]. Permeative bone destruction and periosteal response are observed in osteosarcoma [8].

**Table 1:** Literature review of recurrent cementoblastoma with multifocal growth

Author	Age (years)/Sex	Involved teeth	Initial surgical procedure	Follow-up (years)	Clinical or radiographic features of recurrent lesion
Zaitoun et al. [19] (2007)	10/F	Right mandibular second molar	Enucleation with the extraction of the associated tooth	0.5	Recurrence in one large area and two smaller areas
Ahmad et al. [20] (2014)	14/M	Right mandibular first molar	Enucleation with the extraction of the associated tooth	0.6	Small denticle-like structures
Yoon et al. [5] (2021)	16/M	Right mandibular first molar	Enucleation with the extraction of the associated tooth	4	Multiple cemental masses

The differences between cementum/cementoblast and bone/osteoblast have recently become more apparent. Matthews et al. reported differentially expressed genes between cementoblasts and osteoblasts [22]. They confirmed that the expression of Wnt inhibitors, *Wif1* and *Sfrp1*, and transcription factor, *Barx1*, was elevated in cementoblasts compared to that in osteoblasts [22]. Salmon et al. reported the proteomic analysis of cementum and bone [23], wherein they identified 105 and 83 proteins exclusive to the alveolar bone and dental cementum, respectively. Because tumors tend to resemble their origin, these differences may be applicable to cementoblastoma and osteoblastoma. These studies potentially contribute to the discovery of a marker protein for cementoblastoma and osteoblastoma in the future, although not yet in practical use.

In the present case, the mechanism of showing cellular atypia is unclear; however, there is a similar grouping: pseudomalignant osteoblastoma [24]. The variant contains cells with enlarged hyperchromatic nuclei, which may cause histologic confusion with osteosarcoma. It is not associated with mitotic activity and has no clinical significance; moreover, it has been hypothesized to be degenerative in nature [24]. Atypical cells that comprise clusters were also uncommon findings of cementoblastoma. Although they seemed to be the clustering of macrophages, they were negative for CD68 and partially weakly positive for c-FOS. Therefore, they were presumed to be tumor cells in different differentiation stages. However, this finding is of undetermined significance.

Rearrangement of *FOS* leads to loss of the C-terminal end of c-FOS [12], making the protein resistant to degradation, and results in an intense nuclear immunoreactivity of the truncated c-FOS [12, 13]. In contrast, ubiquitin-independent proteasomal degradation rapidly depletes the wild-type c-FOS [12]. Similar rearrangements of *FOS* were previously found in epithelioid hemangioma [25]. Research indicates that the truncated c-FOS significantly enhances endothelial sprouting in HUVECs through the activation of the Notch signaling pathway and by elevating MMP production [25]. It is inferred that the persistent expression of truncated c-FOS, attributable to its proteasomal degradation resistance, may promote vascular neoplasm development [25]. Although additional research is essential to elucidate the precise role of truncated c-FOS in osteoid osteoma, osteoblastoma, and cementoblastoma, its contribution to tumor growth could be noteworthy [1].

The cause of recurrence has been previously discussed. Brannon et al. reported that recurrence is most likely when curettage is attempted without extraction of associated teeth [3]. Incomplete removal may be a risk of recurrence [5, 26]. Conversely, Chrcanovic et al. argued that preservation of the involved teeth did not appear to influence the recurrence rate. There is a higher probability of lesion recurrence associated with bone expansion and cortical bone perforation [4]. Considering these factors, subtle fractions of tumors may sometimes remain as seeds at the time of enucleation and curettage. Complete tumor fraction removal may be more difficult in larger tumors that tend to show bone expansion or perforation. Thus, recurrence in the present case may be explained by this factor. In the present case, cortical bone expansion and perforation were observed in the primary lesion on CBCT images. It is believed that several tumor cells were left in the surrounding trabecula as a core at the time of enucleation that then formed a multifocal mass.

The interval from initial treatment to recurrence ranged from 4 to 24 months, with a mean interval of 15–16.8 months [3, 4]. However, in the present case, recurrence was observed 60 months after the initial treatment. An earlier diagnosis could have been made with regular follow-ups, requiring less invasive approaches. Thus, our case demonstrates the importance of long-term follow-up of patients with cementoblastoma.

In conclusion, the differential diagnosis for recurrent cementoblastoma includes benign to malignant because of its large size, multicentric growth pattern, and cytological atypia. However, accurate diagnosis can be made in a comprehensive manner considering all histopathological and radiological findings. Pathological analyses of this case suggested that the recurrent event in the cementoblastoma altered their growth pattern and tumor cell shape. In the case of enucleation surgery, long-term follow-up is important because of the risk of recurrence.

## Data Availability

No datasets were generated or analysed during the current study.

## Abbreviations

CBCT: cone-beam computed tomography
HUVECs: human umbilical vein endothelial cells
MMP: matrix metalloproteinase
